# Synthesis and anticancer evaluation of some novel pyrimido[5,4-*e*][1,2,4]triazines and pyrazolo[3,4-*d*]pyrimidine using DMF-DMA as methylating and cyclizing agent

**DOI:** 10.1186/s13065-018-0424-3

**Published:** 2018-05-23

**Authors:** Samar A. El-Kalyoubi

**Affiliations:** 1 Department of Pharmaceutical Organic Chemistry, Faculty of Pharmacy (Girls), Al-Azhar University, Nasr City, Cairo, 11651 Egypt; 2 Department of Medical Chemistry, Faculty of Applied Medical Sciences (Female Section), Jazan University, Jazan, 45142 Saudi Arabia

**Keywords:** 6-Hydrazinyluracil, Pyrimidotriazine, Pyrazolopyrimidine, Dimethylformamide-dimethylacetal, Anticancer activities

## Abstract

**Background:**

Described a series of main target compounds pyrimido[5,4-*e*][1,2,4]triazines is obtained via condensation of 6-hydrazinyluracil with different aromatic aldehydes to give the hydrazones followed by nitrosation with HNO_2_ then intramolecular cyclization. On the other hand, pyrazolopyrimidines can be obtained by the reaction of hydrazones with dimethylformamide-dimethylacetal (DMF-DMA), DMF-DMA in the presence of DMF or by refluxing the hydrazinyluracil with DMF-DMA in the presence of DMF directly. The newly synthesized compounds are evaluated in vitro for their anticancer activity against human lung carcinoma (A549).

**Results:**

A newly substituted compounds of benzaldehyde-pyrimidin-4-yl)hydrazones (**5a**–**f**), pyrimido[5,4-*e*][1,2,4]triazines **6a**–**e**, arylethylidenehydrazinylpyrimidine **7a**,**b** and pyrazolopyrimidines **9**,**11** are screened for cytotoxic activity against human lung carcinoma (A549) cell line. They exhibited a good yield. Compound **6b** shows the highest effect with IC_50_ value 3.6 μM, followed by compounds **9**, **5a**, **8**, **5e**, **6e**, **5b**, **5f**, **7a**, **5c**, **6c**, **7b**, **6a**, **11**, **5d** and **6d**.

**Conclusion:**

A simple and efficient route is used for the synthesis of pyrimido[5,4-*e*][1,2,4]triazines and pyrazolopyrimidines. The synthesized compounds are screened for antitumor activity. 
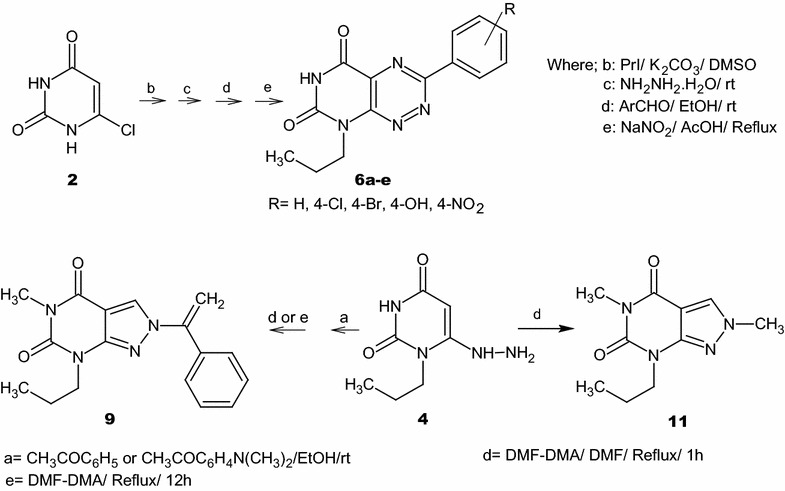

## Background

Triazine is analogues of six membered benzene ring via replacing the three carbon atoms with nitrogens. 1,2,4-triazine and their fused ring structures with one or more heterocycles represent an important class of nitrogen heterocycles compounds. It possess the motif part of naturally and synthetic pharmaceutical products [1–9]. They exhibit a broad spectrum biological effects [10] with antibacterial [11, 12], antitumor [13, 14], anticonvulsant [15], anti-inflammatory [16], and antiviral properties [17]. 6-Azacytosine and 6-azauracil are used as effective antiviral and antitumor activities [18–21]. The tirapazamine (TPZ) is efficacious in the treatment of different human cancer cells via inducing DNA damage in poorly oxygenated tumor cells [22]. 

The pyrimidotriazine antibiotics represent a wide spectrum of both antimicrobial and antitumor activities [23]. Pyrimido[5,4-*e*][1,2,4]triazine constitutes the essential active ingredient of the antibiotics like fervenulin (which is formed from actinomyces), xanthothricin, and reumycin [2, 3]. Reumycin [24] is isolated from actinomyces rectus bruneus and used as an antitumor antibiotic for treating brain tumors. Other hetero annelated 1,2,4-triazines have clinical antiviral effect against influenza A and B viruses [4], anti-HIV and anticancer activity [5, 6]. They also show antimicrobial, antifungal effects and cytotoxicity to MCF-7 cells [7, 8]. Fervenulin (planomycin), and its tautomeric isomer toxoflavin (panthothricin) {1.6-dimethylpyrimido[5,4-e][1,2,4]triazine-5,7(1H,6H)-dione} reveal a wide spectrum antibacterial, antifungal, herbicidal and anticancer activities [25–27]. 

Pyrazolopyrimidines constitute the core of many drugs with wide variety of applications in the field of medicine. They are bioactive isomeric purine analogues and have a significant activity as antimetabolites in purine biochemical reactions [28–31]. They have diverse pharmacological effect as tuberculostatic [32], antimicrobial activities [33], neuroleptic [34], CNS depressant [32], antihypertensive [35] and antileishmanial [36].

In this regard, our strategy is directed towards the synthesis of new pyrimido[5,4-*e*][1,2,4]triazine and pyrazolopyrimidine compounds because they have found wide applications in pharmaceutical fields. The structure of newly synthesized pyrimidotriazines and pyrazolopyrimidines are proven on the basis of their ^1^H-NMR, mass spectral data, IR and elemental analysis.

## Result and discussion

### Chemistry

**Scheme 1:**
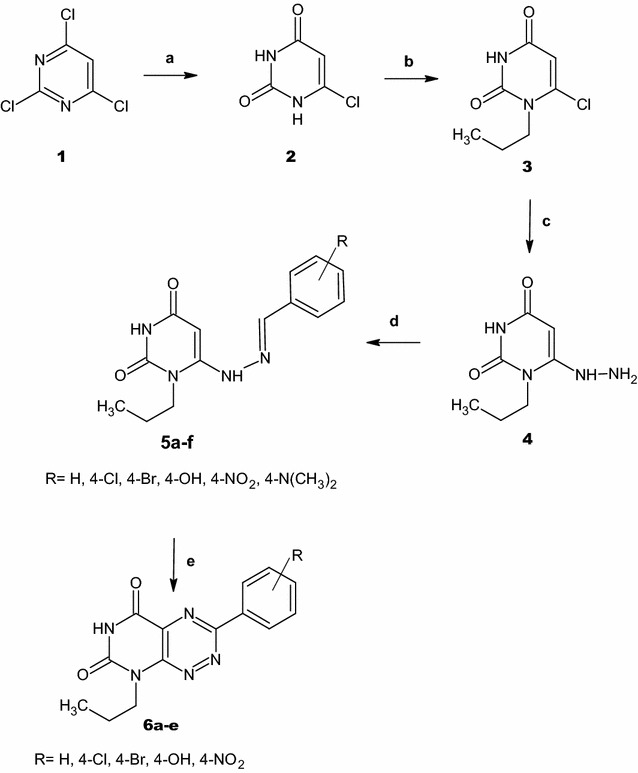
Reaction of 6-hydrazinyluracil with different aromatic aldehydes and formation of pyrimidotriazines. a = NaOH/H_2_O/Reflux; b = PrI/K_2_CO_3_/DMSO; c = NH_2_NH_2_.H_2_O/rt; d = ArCHO/EtOH/rt; e = NaNO_2_/AcOH/Reflux

In continuation to our research, the importance of fervenulin and its diverse pharmacological activity on the medical field, especially as antitumors, we became interested in the prospect of developing our strategies to synthesize new fervenulin analogues of pyrimidotriazine and pyrazolopyrimidine derivatives using 6-hydrazinyl-1-propyluracil (**4**) as a core for construction. This substrate is prepared via simple hydrolysis of 2,4,6-trichloropyrimidine [37] followed by N-1 selective alkylation using propyl iodide in DMSO in the presence of potassium carbonate as a basic medium [38] then hydrazinolysis of 6-chloro-1-propyluracil (**3**) with hydrazine hydrate [38–40]. Condensation of substrate **4** with different aromatic aldehydes in ethanol at room temperature for 1 h leads to the formation of hydrazones **5a**–**f** in a good yield (Scheme 1).

The IR spectra of hydrazones displayed the N–H stretching bands at 3271–3122 cm^−1^. The stretching band of the two C=O groups (*Amide I*) is displayed within the range 1740–1625 cm^−1^. Compound **5d** showed O–H stretching bands at 3560 cm^−1^ while the nitro group in compound **5e** shows strong asymmetric and symmetric NO_2_ stretching bands at 1514 and 1337 cm^−1^, respectively. The ^1^H-NMR spectra supported the previous observation from the IR spectra, where N3–H and C6–NH is highly deshielded. They appeared around *δ* 10.75–10.05 ppm, while the α-CH of hydrazone appeared at the range *δ* 8.48–8.24 ppm. The C5-H was the most shielded as expected around *δ* 5.46–5.30 ppm. The downfield shift of the α-carbon of hydrazone appeared around *δ* 145 ppm in the ^13^C-NMR spectra.

**Scheme 2:**
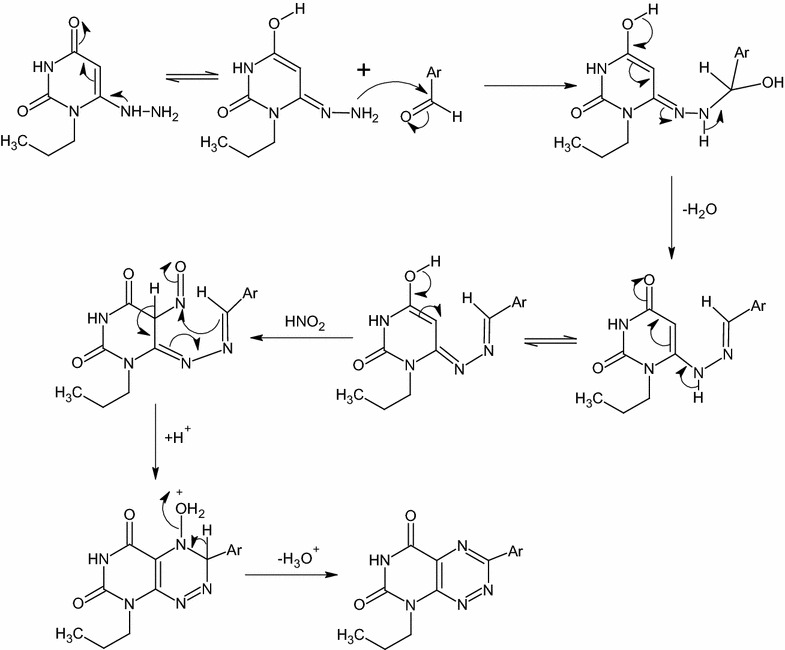
The plausible reaction mechanism formation of compounds **5a**–**f** and **6a**–**e**

Pyrimidotriazines **6a**–**e** is isolated by the nitrosation of hydrazone compounds **5a**–**e** at C-5 with in situ prepared nitrous acid. The inseparable 5-nitroso-derivatives undergoes cyclization via the nucleophilic attack of the electron rich α-carbon of the hydrazones on the nitroso group to form hydroxylamine intermediates, which are converted into the target pyrimidotriazines **6a**–**e** by protonation of the *N*-hydroxyl group followed by the elimination of H_3_O^+^ (Scheme 2). The IR spectra of **6a**–**e** displayed broad absorption bands of NH stretching in the region of 3180–3135 cm^−1^. The two bands of C=O groups gave rise in the region of 1725–1670 cm^−1^.

Moreover, the cyclization of the hydrazone series are confirmed in ^1^H-NMR spectra through the disappearance of both the α-CH hydrazone at *δ* 8.48–8.24 ppm and the C5-H of uracil at *δ* 5.46–5.30 ppm.

**Scheme 3:**
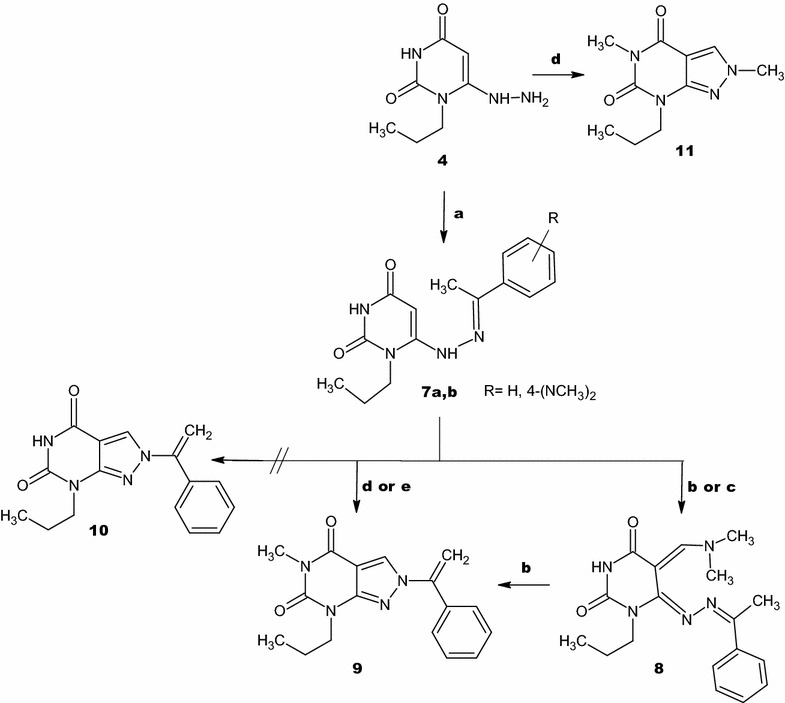
Synthesis of pyrazolopyrimidines. a = EtOH/rt; b = DMF-DMA/Reflux/1 h; c = DMF-DMA/DMF/Reflux/15 min; d = DMF-DMA/DMF/Reflux/1 h; e = DMF-DMA/Reflux/12 h

Another condensation reaction is obtained via condensation of **4** with different acetophenones by stirring at room temperature for 3–4 h (Scheme 3). The IR spectra of **7a**,**b** displayed stretching bands at the range of 3188–3154 cm^−1^ due to N–H absorption and characteristic bands at the range 1715–1691 cm^−1^ due to absorption of C=O groups. The mass spectra of these compounds show the expected molecular ions, whereas their ^1^H-NMR spectra exhibited two signals at *δ* 11.11–11.06 ppm and at *δ* 9.13–8.96 ppm ascribed for N3–H and C6–NH protons respectively. The singlet signals of the methyl group protons at the α-carbon appeared at δ 2.42–2.37 ppm while for the CH-5 position appeared at δ 5.48–5.39 ppm. ^13^C-NMR confirmed the structure of **7a**,**b** where the key signals at δ 78.8–79.8 ppm and δ 14.3–14.2 ppm are assigned to *sp*^2^ carbon at position 5 and *sp*^3^ carbon attached to the α-carbon respectively.

**Scheme 4:**
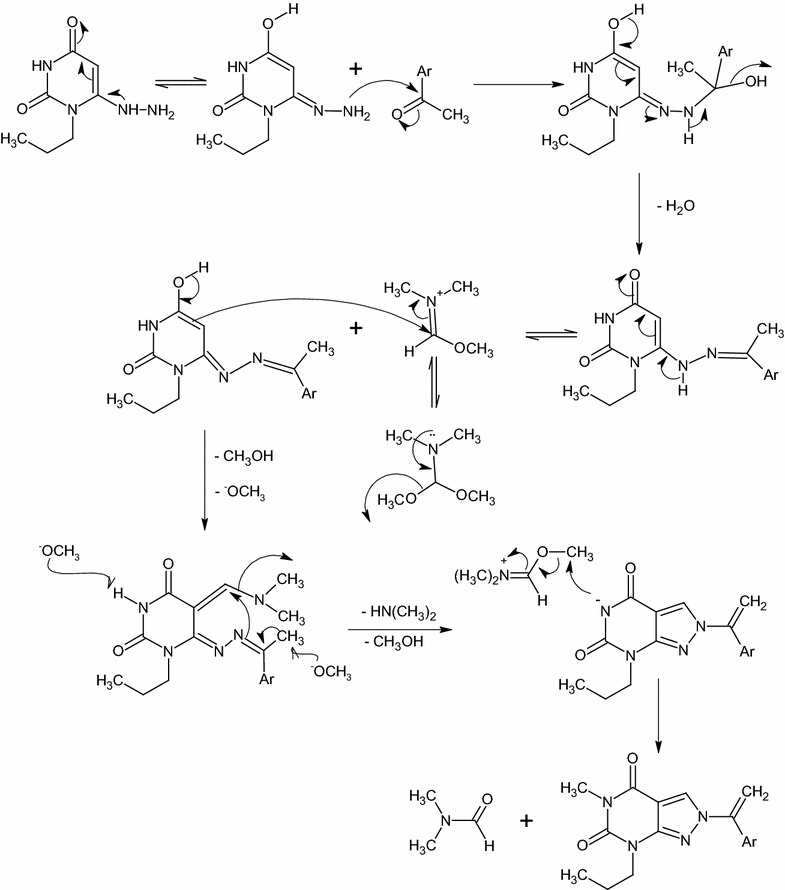
The plausible reaction mechanism formation of compounds **7a**,**b** and the intermediate **8** and **9**

The target compound **9** is prepared by refluxing of **7a** with DMF-DMA for 12 h or DMF-DMA in presence of DMF as a solvent for 1 h (Scheme 3). DMF-DMA is a convenient electrophile to introduce one-carbon units. The reaction proceeds via nucleophilic attack of C-5 to electrophilic carbon center of acetal in DMF-DMA followed by intramolecular cyclization and elimination of dimethylamine. A subsequent methylation of NH-5 is observed which arises from O–CH_3_ group of the acetal not N–CH_3_ as illustrated in Scheme 4. The plausible mechanism is proved by isolation of the intermediate **8**. This intermediate is easily identified in IR, Mass, ^1^H-NMR and ^13^C-NMR spectra. A broad stretching absorption band of NH appears at the region of 3136 cm^−1^ of the intermediate **8** in IR spectra and disappears in the target compound **9**. Furthermore, ^1^H-NMR showed the disappearance of CH-5 proton at δ 5.48–5.39 ppm and the appearance of a singlet signal at δ 8.08 ppm characteristic for CH–N proton, a singlet signal of α-C–CH_3_ at δ 2.39 ppm and two characteristic N–CH_3_ group at δ 3.19, 3.05 ppm of compound **8** and disappears in compound **9** due to the elimination of dimethylamine (Scheme 4).

Whereas, the alkylation on N-5 in compound **9** is proven without doubt by the disappearance of a singlet signal of NH-5 proton at δ 10.13 ppm and the appearance of the *sp*^3^ singlet signal at δ 3.23 ppm characteristic of CH_3_ appears in ^1^H-NMR and a signal at δ 27.6 ppm in ^13^C-NMR. In addition, the ^1^H-NMR shows a singlet signal of CH-3 at δ 8.60 ppm and two doublet signals at δ 5.71–5.61 ppm corresponding to the two protons of methylene group which indicates that they are not magnetically equivalent. ^13^C-NMR shows the appearance of signals at δ 134.2 and 101.7 ppm characteristic for C-3 and methylene carbon atom respectively.

**Scheme 5:**
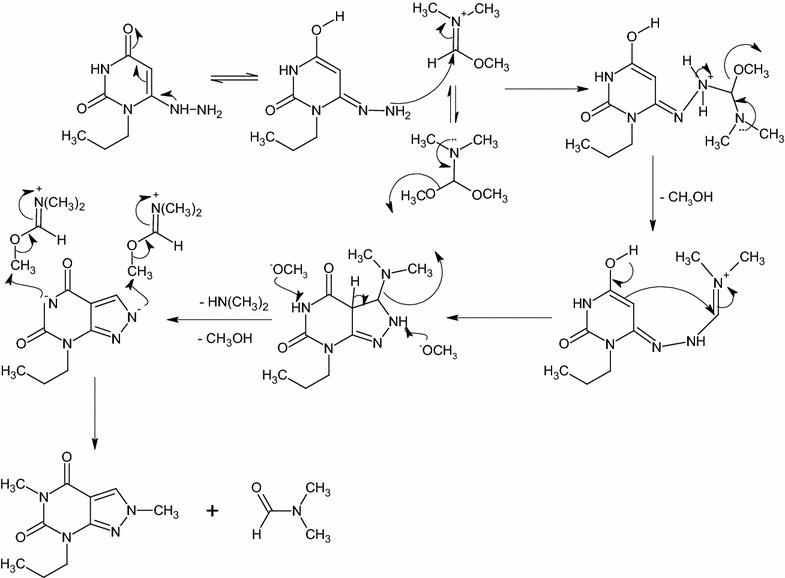
The plausible reaction mechanism formation of compound **11**

Treatment of **4** with DMF-DMA in the presence of DMF by refluxing for 1 h yielded compound **11** (Scheme 3). IR spectra shows characteristic absorption band at 1751, 1698 cm^−1^ for C=O groups. ^1^H-NMR spectrum displays three singlet signals at δ 8.41, 3.88 and 3.19 ppm for CH-3, N(2)–CH_3_ and N(5)–CH_3_ respectively. On the other hand, ^13^C-NMR showed C–N(2) at δ 40.4 ppm and C–N(5) at δ 27.5 ppm which confirms the alkylation of N(5) with DMF-DMA. The plausible mechanism for this reaction is shown in (Scheme 5).

## Biological investigation

### Cytotoxic activity

**Fig. 1:**
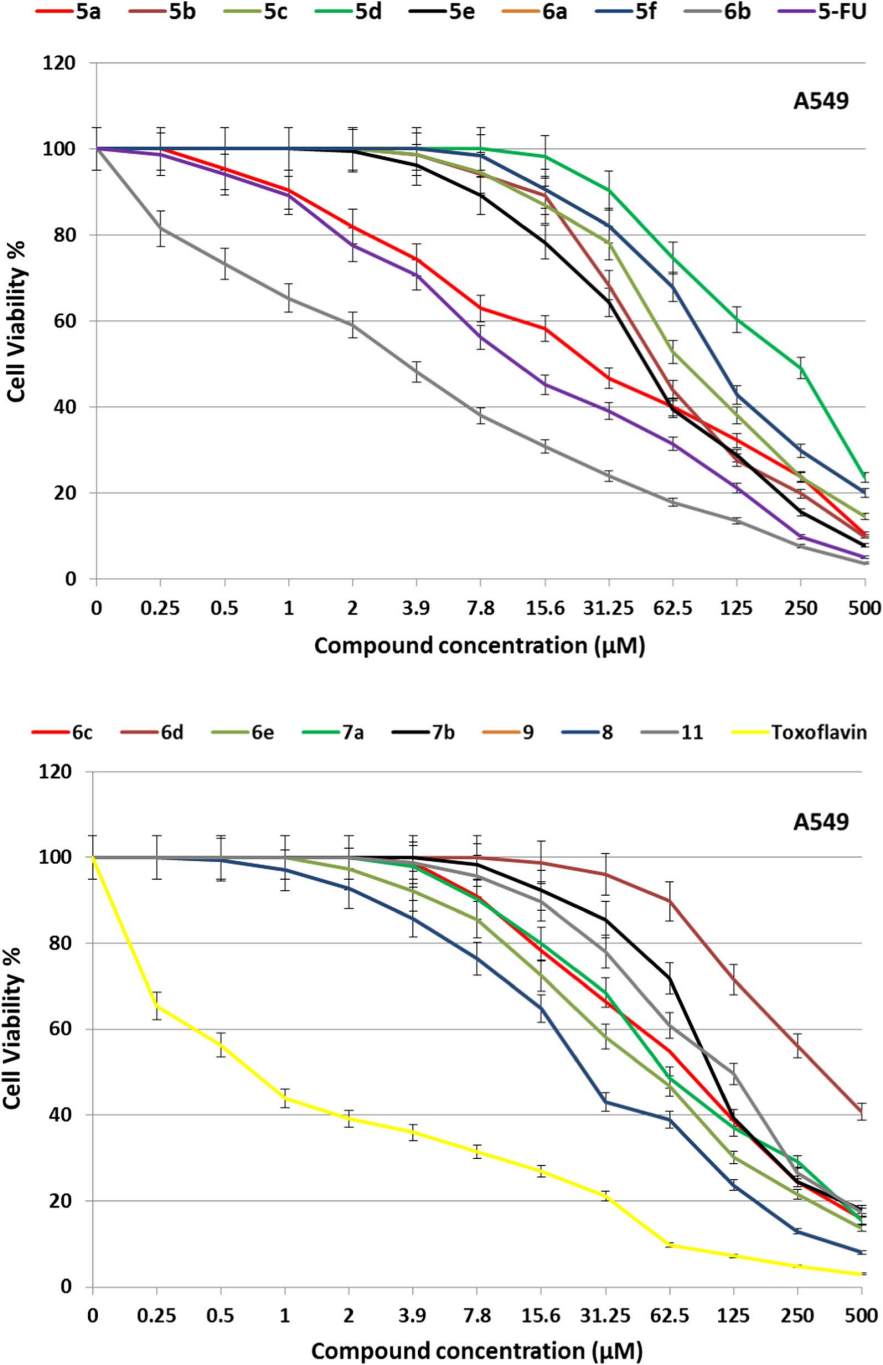
Growth inhibition curves showing A549 cell line treated with the tested compounds at different concentrations compared with reference drugs 5-flourouracil and toxoflavin

The in vitro growth inhibitory rates against human lung carcinoma (A549) cell line and effective antitumor doses (as measured by IC_50_) of the synthesized compounds are investigated in comparison with the well-known anticancer standard drugs toxoflavin and 5-fluorouracil, using crystal violet colorimetric viability assay. Data generated are used to plot dose response curves and presented in Table 1 and Fig. 1. The results reveal that all the tested compounds show high variation in the inhibitory growth rates and activities to the tumor cell line in a concentration dependent manner as shown in (Table 1).

**Table 1:** The IC_50_ values represent the compound concentration (μM) required to inhibit A549 tumor cell proliferation by 50%

Compounds	IC_50_ (µM)	Compounds	IC_50_ (µM)
**5a**	26.8 ± 1.3	**6c**	81.5 ± 4.3
**5b**	54.7 ± 2.1	**6d**	379.4 ± 24.8
**5c**	74.3 ± 5.1	**6e**	53.8 ± 3.5
**5d**	238.7 ± 12.5	**7a**	60.5 ± 2.6
**5e**	49.3 ± 4.1	**7b**	104.6 ± 4.8
**5f**	60.2 ± 3.2	**8**	28.4 ± 1.6
**6a**	107.1 ± 6.2	**9**	26.3 ± 0.9
**6b**	3.6 ± 0.2	**11**	123 ± 6.1
Toxoflavin^a^	0.7 ± 0.1	^a^5-FU	10.5 ± 0.1

^a^Reference drugs; *5-FU* (5-fluorouracil)

From the results in Fig. 1, it is clear that all the tested compounds are found to be very active at 500 μM against human lung carcinoma (A549) cell line after treatment for 72 h with inhibition ratio values between 60 and 97%. The difference between inhibitory activities of all compounds with different concentrations is statistically significant (p  < 0.001).

The highest activity against human lung carcinoma (A549) cell line is measured for compound **6b** with IC_50_ value 3.6 μM, followed by compounds **9**, **5a**, **8**, **5e**, **6e**, **5b**, **5f**, **7a**, **5c**, **6c**, **7b**, **6a**, **11**, **5d** and **6d** with IC_50_ values of 26.3, 26.8, 28.4, 49.3, 53.8, 54.7, 60.2, 60.5, 74.3, 81.5, 104.6, 107.1, 123, 238.7, and 379.4 μM, compared with reference drugs 5-fluorouracil (10.5 μM) and toxoflavin (0.7 μM).

## Methods

### Instruments

All melting points were determined with an electrothermal melting-temperature II apparatus and are uncorrected. Element analyses are performed at the regional center for mycology and biotechnology at Al-Azhar University. The infrared (IR) spectra are recorded using potassium bromide disc technique on Nikolet IR 200 FT IR. Mass spectra are recorded on a DI-50 unit of Shimadzu GC/MS-QP 5050A at the regional center for mycology and biotechnology at Al-Azhar University. ^1^H-NMR and ^13^C-NMR spectra are determined on Bruker 400 MHz spectrometer using DMSO-d_6_ as a solvent, applied nucleic acid research center, Zagazig University, Egypt. All reactions are monitored by TLC using precoated plastic sheets silica gel (Merck 60 F_254_). Spots are visualized by irradiation with UV light (254 nm). The used solvent system is chloroform: methanol (9:1) and ethyl acetate: toluene (1:1).

### Synthesis

6-Chlorouracil (**2**) was prepared according to the reported method [37].

6-Chloro-1-propyluracil (**3**) was prepared according to the reported method [38].

6-Hydrazinyl-1-propyluracil (**4**) [38–40].

#### 4-Substituted benzaldehyde(2,6-dioxo-3-propyl-1,2,3,6-tetrahydropyrimidin-4-yl)hydrazones (**5a**–**f**)

A mixture of 6-hydrazinyl-1-propyluracil (**4**) (2.17 mmol) and appropriate benzaldehydes (2.17 mmol) in ethanol (25 mL) is stirred at room temperature for 1 h. The formed precipitate is collected by filtration, washed with ethanol and crystallized from ethanol.

#### Benzaldehyde(2,6-dioxo-3-propyl-1,2,3,6-tetrahydropyrimidin-4-yl)hydrazone (**5a**)

Yield: 83%; m.p. = 218–219 °C; IR (KBr) ν_max_ (cm^−1^): 3224 (NH), 3045 (CH arom.), 2969, 2908 (CH aliph.), 1739, 1647 (C=O), 1550 (C=N), 1516 (C=C); ^1^H-NMR (DMSO-*d*_6_): 10.69 (s, 1H, NH), 10.38 (s, 1H, NH), 8.39 (s, 1H, CH), 7.73–7.71(d, 2H, *J *= 7.6 Hz, H_arom_), 7.45–7.42 (m, 3H, arom.), 5.38 (s, 1H, CH-5), 3.89–3.85 (t, 2H, CH_2_), 1.60–1.55 (m, 2H, CH_2_), 0.91–0.87 (t, 3H, CH_3_); ^13^C-NMR (DMSO-*d*_6_): δ = 162.4, 152.4, 151.0, 146.5, 134.0, 130.0, 128.9, 126.9, 77.2, 42.2, 21.0, 10.7 ppm; MS: *m*/*z* (%) = M^+^, 272 (83), 243 (61), 216 (36), 153 (36), 145 (31), 144 (25), 110 (27), 106 (58), 104 (100), 103 (22), 90 (38), 89 (33), 77 (52); Anal. calcd. for C_14_H_16_N_4_O_2_ (272.30): C, 61.75; H, 5.92; N, 20.58. Found: C, 61.86; H, 5.97; N, 20.73.

#### 4-Chlorobenzaldehyde(2,6-dioxo-3-propyl-1,2,3,6-tetrahydropyrimidin-4-yl)hydrazone (**5b**)

Yield: 85%; m.p. = 238–239 °C; IR (KBr) ν_max_ (cm^−1^): 3224 (NH), 3056 (CH arom.), 2936 (CH aliph.), 1700, 1630 (C=O), 1594 (C=N), 1558 (C=C), 870 (*p*-substituted phenyl); ^1^H-NMR (DMSO-*d*_6_): 10.68 (s, 1H, NH), 10.42 (s, 1H, NH), 8.37 (s, 1H, CH), 7.75–7.73 (d, 2H, *J* = 8.4 Hz, H_arom_), 7.51–7.49 (d, 2H, *J* = 8.4 Hz, H_arom_), 5.39 (s, 1H, CH-5), 3.89–3.85 (t, 2H, CH_2_), 1.60–1.54 (m, 2H, CH_2_), 0.90–0.87 (t, 3H, CH_3_); ^13^C-NMR (DMSO-*d*_6_): δ = 162.4, 152.4, 151.0, 145.1, 134.4, 133.0, 129.0, 128.5, 77.4, 42.2, 21.0, 10.7 ppm; MS: *m*/*z* (%) = M + 2, 308 (31), M^+^, 306 (100), 279 (30), 277 (96), 252 (22), 250 (64), 179 (31), 178 (27), 154 (12), 153 (52), 152 (39), 142 (23), 140 (82), 139 (33), 138 (86), 136 (63), 127 (27), 125 (17), 124 (25), 113 (22), 111 (48), 110 (37); Anal. calcd. for C_14_H_15_ClN_4_O_2_ (306.74): C, 54.82; H, 4.93; N, 18.26. Found: C, 55.04; H, 5.01; N, 18.43.

#### 4-Bromobenzaldehyde(2,6-dioxo-3-propyl-1,2,3,6-tetrahydropyrimidin-4-yl)hydrazone (**5c**)

Yield: 84%; m.p. = 242–243 °C; IR (KBr) ν_max_ (cm^−1^): 3122 (NH), 3028 (CH arom.), 2971 (CH aliph.), 1740, 1647 (C=O), 1548 (C=N), 1515 (C=C), 869 (*p*-substituted phenyl); ^1^H-NMR (DMSO-*d*_6_): 10.68 (s, 1H, NH), 10.43 (s, 1H, NH), 8.36 (s, 1H, CH), 7.70–7.63 (m, 4H, arom.), 5.39 (s,1H, CH-5), 3.89–3.85 (t, 2H, CH_2_),1.60–1.54 (m, 2H, CH_2_), 0.90–0.84 (t, 3H, CH_3_); ^13^C-NMR (DMSO-*d*_6_): δ = 162.5, 152.4, 151.0, 145.2, 133.3, 131.9, 128.7, 123.2, 77.4, 42.2, 21.0, 10.7 ppm; MS: *m*/*z* (%) = M + 2, 353 (6), M^+^, 351 (12), 350 (77), 323 (23), 321 (68), 296 (61), 294 (100), 186 (50), 183 (48), 182 (47), 181 (44), 168 (30), 157 (36), 154 (20), 253 (27), 152 (56), 144 (41), 140 (33), 110 (31), 102 (23), 89 (47), 76 (41); Anal. calcd. for C_14_H_15_BrN_4_O_2_ (351.20): C, 47.88; H, 4.3; N, 15.95. Found: C, 48.02; H, 4.28; N, 16.02.

#### 4-Hydroxybenzaldehyde(2,6-dioxo-3-propyl-1,2,3,6-tetrahydropyrimidin-4-yl)hydrazone (**5d**)

Yield: 78%; m.p. = 213–214 °C; IR (KBr) ν_max_ (cm^−1^): 3560 (OH), 3271 (NH), 3020 (CH arom.), 2968 (CH aliph.), 1705, 1625 (C=O), 1581 (C=N), 1512 (C=C), 834 (*p*-substituted phenyl); ^1^H-NMR (DMSO-*d*_6_): 10.58 (s, 1H, NH), 10.15 (s, 1H, NH), 9.90 (s, 1H, OH), 8.28 (s, 1H, CH), 7.56–7.53 (d, 2H, *J *= 8.4 Hz, H_arom_), 6.84–6.82 (d, 2H, *J *= 8.4 Hz, H_arom_), 5.32 (s, 1H, CH-5), 3.87 - 3.85 (t, 2H, CH_2_),1.59–1.57 (m, 2H, CH_2_), 0.90–0.87 (t, 3H, CH_3_); ^13^C-NMR (DMSO-*d*_6_): δ = 162.5, 159.4, 152.5, 151.1, 147.0, 128.7, 125.1, 115.8, 76.6, 42.1, 21.0, 10.7 ppm; MS: *m*/*z* (%) = M^+^, 288 (40), 259 (21), 232 (31), 161 (72), 160 (48), 146 (24), 122 (61), 121 (26), 120 (88), 119 (100), 106 (30), 1105 (20); Anal. calcd. for C_14_H_16_N_4_O_3_ (288.30): C, 58.32; H, 5.59; N, 19.43. Found: C, 58.50; H, 5.67; N, 19.61.

#### 4-Nitrobenzaldehyde(2,6-dioxo-3-propyl-1,2,3,6-tetrahydropyrimidin-4-yl)hydrazone (**5e**)

Yield: 91%; m.p. = 228–230 °C; IR (KBr) ν_max_ (cm^−1^): 3150 (NH), 3022 (CH arom.), 2968 (CH aliph.), 1740, 1692 (C=O), 1593 (C=N), 1561 (C=C), 1514 (NO_2*asymstr*_), 1337 (NO_2*symstr*_), 843 (*p*-substituted phenyl); ^1^H-NMR (DMSO-*d*_6_): 10.75 (s, 1H, NH), 10.68 (s, 1H, NH), 8.48 (s, 1H, CH), 8.28–8.26 (d, 2H, *J* = 7.2 Hz, H_arom_), 7.99–7.97 (d, 2H, *J *= 7.2 Hz, H_arom_), 5.46 (s, 1H, CH-5), 3.90–3.87 (t, 2H, CH_2_), 1.61–1.55 (m, 2H, CH_2_), 0.91–0.87 (t, 3H, CH_3_); ^13^C-NMR (DMSO-*d*_6_): δ = 162.4, 152.3, 151.0, 147.7, 143.7, 140.3, 127.8, 124.1, 78.1, 42.3, 21.1, 10.7 ppm; MS: *m*/*z* (%) = M^+^, 317 (39), 288 (100), 261 (83), 190 (38), 168 (22), 153 (91), 152 (54), 151 (34), 149 (33), 127 (41), 110 (48), 103 (34), 89 (60), 84 (31), 76 (33), 68 (50); Anal. calcd. for C_14_H_15_N_5_O_4_ (317.30): C, 52.99; H, 4.76; N, 22.07. Found: C, 53.15; H, 4.83; N, 22.24.

#### 4-(Dimethylamino)benzaldehyde(2,6-dioxo-3-propyl-1,2,3,6-tetrahydro-pyrimidin-4-yl)hydrazone (**5f**)

Yield: 76%; m.p. = 234–235 °C; IR (KBr) ν_max_ (cm^−1^): 3220 (NH), 3045 (CH arom.), 2958, 2869 (CH aliph.), 1729, 1693 (C=O), 1593 (C=N), 1519 (C=C), 855 (*p*-substituted phenyl); ^1^H-NMR (DMSO-*d*_6_): 10.54 (s, 1H, NH), 10.05 (s, 1H, NH), 8.24 (s, 1H, CH), 7.53–7.51 (d, 2H, *J* = 8.8 Hz, H_arom_), 6.76–6.74 (d, 2H, *J *= 8.8 Hz, H_arom_), 5.30 (s, 1H, CH-5), 3.87–3.83 (t, 2H, CH_2_), 2.97 (s, 6H, 2 CH_3_), 1.59–1.54 (m, 2H, CH_2_), 0.90–0.87 (t, 3H, CH_3_); ^13^C-NMR (DMSO-*d*_6_): δ = 162.4, 158.1, 154.0, 151.9, 147.6, 128.2, 121.3, 111.8, 76.3, 42.0, 41.1, 21.0, 10.7 ppm; MS: *m*/*z* (%) = M^+^, 315 (100), 314 (10), 259 (8), 202 (11), 188 (21), 173 (46), 148 (49), 147 (61), 146 (50), 145 (43), 133 (39), 132 (30); Anal. calcd. for C_16_H_21_N_5_O_2_ (315.37): C, 60.94; H, 6.71; N, 22.21. Found: C, 61.23; H, 6.83; N, 22.37.

#### 3-Aryl-8-propylpyrimido[5,4-*e*][1,2,4]triazine-5,7(6*H*,8*H*)-diones (**6a**–**e**)

A solution of 4-substituted benzaldehyde(2,6-dioxo-3-propyl-1,2,3,6-tetrahydropyrimidin-4-yl)hydrazones (**5a**–**e**) (0.98 mmol) in glacial acetic acid (4 mL) is treated with sodium nitrite (1.16 mmol) by heating under reflux for 3–4 h. The reaction mixture is evaporated under reduced pressure. The residue is treated with ethanol (10 mL); the formed precipitate is filtered, washed with ethanol, and crystallized from DMF/ethanol (1:2) to afford compounds **6a**–**e**.

#### **3**-Phenyl-8-propylpyrimido[5,4-*e*][1,2,4]triazine-5,7(6*H*,8*H*)-dione (**6a**)

Yield: 71%; m.p. = 290–291 °C; IR (KBr) ν_max_ (cm^−1^): 3173 (NH), 3024 (CH arom.), 2968, 2840 (CH aliph.), 1716, 1670 (C=O), 1565 (C=N), 1535 (C=C); ^1^H-NMR (DMSO-*d*_6_): 12.25 (s, 1H, NH), 8.42–8.40 (d, 2H, *J* = 5.2 Hz, H_arom_), 7.62–7.61 (m, 3H, arom.), 3.24–3.21 (t, 2H, CH_2_), 1.68–1.63 (m, 2H, CH_2_), 0.98–0.94 (t, 3H, CH_3_); ^13^C-NMR (DMSO-*d*_6_): δ = 160.4, 154.7, 150.8, 149.4, 146.2, 134.2, 131.3, 129.3, 127.1, 42.4, 20.6, 11.0 ppm; MS: *m*/*z* (%) = M^+^, 283 (16), 255 (20), 254 (38), 213 (17), 171 (13), 105 (100), 104 (13), 103 (10), 77 (29); Anal. calcd. for C_14_H_13_N_5_O_2_ (283.28): C, 59.36; H, 4.63; N, 24.72. Found: C, 59.58; H, 4.71; N, 24.89.

#### 3-(4-Chlorophenyl)-8-propylpyrimido[5,4-*e*][1,2,4]triazine-5,7(6*H*,8*H*)-dione (**6b**)

Yield: 78%; m.p. = 258–260 °C; IR (KBr) ν_max_ (cm^−1^): 3135 (NH), 3034 (CH arom.), 2972, 2839 (CH aliph.), 1725, 1672 (C=O), 1586 (C=N), 1553 (C=C), 841 (*p*-substituted phenyl); ^1^H-NMR (DMSO-*d*_6_): 12.32 (s, 1H, NH), 8.42–8.40 (d, 2H, *J* = 8.4 Hz, H_arom_), 7.69–7.67 (d, 2H, *J* = 8.4 Hz, H_arom_), 4.25–4.21 (t, 2H, CH_2_), 1.76–1.71 (m, 2H, CH_2_), 0.98–0.94 (t, 3H, CH_3_); ^13^C-NMR (DMSO-*d*_6_): δ = 160.2, 158.1, 150.8, 149.3, 136.2, 134.4, 134.0, 129.3, 128.6, 42.9, 20.3, 11.0 ppm; MS: *m*/*z* (%) = M + 2, 319 (6), M^+^, 317 (17), 290 (24), 289 (26), 288 (64), 249 (14), 247 (40), 141 (31), 139 (100),; Anal. calcd. for C_14_H_12_ClN_5_O_2_ (317.73): C, 52.92; H, 3.81; N, 22.04. Found: C, 53.14; H, 3.87; N, 22.27.

#### 3-(4-Bromophenyl)-8-propylpyrimido[5,4-*e*][1,2,4]triazine-5,7(6*H*,8*H*)-dione (**6c**)

Yield: 76%; m.p. = 250–252 °C; IR (KBr) ν_max_ (cm^−1^): 3180 (NH), 3084 (CH arom.), 2809 (CH aliph.), 1712, 1675 (C=O), 1579 (C=N), 1545 (C=C) 845 (*p*-substituted phenyl); ^1^H-NMR (DMSO-*d*_6_): 12.22 (s, 1H, NH), 8.35–8.33 (d, 2H, *J* = 8.8 Hz, H_arom_), 7.84–7.82 (d, 2H, *J* = 8.8 Hz, H_arom_), 4.25–4.21 (t, 2H, CH_2_), 1.77–1.71 (m, 2H, CH_2_), 0.98–0.94 (t, 3H, CH_3_); ^13^C-NMR (DMSO-*d*_6_): δ = 160.0, 158.2, 150.8, 149.1, 134.5, 132.3, 131.3, 129.0, 125.2, 42.9, 20.3, 11.0 ppm; MS: *m*/*z* (%) = M + 2, 364 (2), M^+^, 362 (3), 258 (29), 257 (15), 256 (15), 254 (24), 222 (100), 202 (31), 188 (20), 187 (22), 164 (22), 163 (21), 121 (24), 69 (64), 44 (78), 40 (53); Anal. calcd. for C_14_H_12_BrN_5_O_2_ (362.18): C, 46.43; H, 3.34; N, 19.34. Found: C, 46.71; H, 3.39; N, 19.51.

#### 3-(4-Hydroxyphenyl)-8-propylpyrimido[5,4-*e*][1,2,4]triazine-5,7(6*H*,8*H*)-dione (**6d**)

Yield: 82%; m.p. = 236–238 °C; IR (KBr) ν_max_ (cm^−1^): 3170 (NH), 3026 (CH arom.), 2833 (CH aliph.), 1722, 1677 (C=O), 1558 (C=N), 1537 (C=C), 846 (*p*-substituted phenyl); ^1^H-NMR (DMSO-*d*_6_): 12.22 (s, 1H, NH), 8.24–8.22 (d, 2H, *J* = 8.4 Hz, H_arom_), 6.74–6.72 (d, 2H, *J* = 8.4 Hz, H_arom_), 3.79–3.76 (t, 2H, CH_2_), 1.69–1.63 (m, 2H, CH_2_), 0.89–0.85 (t, 3H, CH_3_); MS: *m*/*z* (%) = M^+^, 299 (17), 271 (22), 256 (20), 223 (34), 222 (40), 221 (100), 105 (60), 100 (94), 98 (76), 84 (33), 83 (29), 77 (29), 69 (31), 57 (26); Anal. calcd. for C_14_H_13_N_5_O_3_ (299.28): C, 56.18; H, 4.38; N, 23.40. Found: C, 56.34; H, 4.47; N, 23.62.

#### 3-(4-Nitrophenyl)-8-propylpyrimido[5,4-*e*][1,2,4]triazine-5,7(6*H*,8*H*)-dione (**6c**)

Yield: 70%; m.p. = 267–268 °C; IR (KBr) ν_max_ (cm^−1^): 3165 (NH), 3067 (CH arom.), 2974, 2811 (CH aliph.), 1720,1702 (C=O), 1606 (C = N), 1559 (C=C), 1518 (NO_2*asymstr*_),1346 (NO_2*symstr*_), 844 (*p*-substituted phenyl); ^1^H-NMR (DMSO-*d*_6_): 12.31 (s, 1H, NH), 8.66–8.62 (d, 2H, *J* = 9.2 Hz, H_arom_), 8.46–8.43 (d, 2H, *J* = 9.2 Hz, H_arom_), 4.25–4.21 (t, 2H, CH_2_), 1.75–1.70 (m, 2H, CH_2_), 0.98–0.94 (t, 3H, CH_3_); ^13^C-NMR (DMSO-*d*_6_): δ = 162.0, 156.9, 151,4, 151.2, 148.9, 140.4, 134.2, 128.2, 124.4, 42.8, 20.4, 11.1 ppm; MS: *m*/*z* (%) = M^+^, 328 (11), 300 (18), 299 (63), 258 (32), 244 (21), 151 (42), 150 (100), 104 (18), 76 (20), 65 (21), 43 (23); Anal. calcd. for C_14_H_13_N_5_O_3_ (328.28): C, 51.22; H, 3.68; N, 25.60. Found: C, 51.37; H, 3.65; N, 25.81.

#### 6-[2-(1-Arylethylidene)hydrazino]-1-propylpyrimidine-2,4(1*H*,3*H*)-diones (**7a**,**b**)

A mixture of 6-hydrazinyl-1-propyluracil (**4**) (2.72 mmol) and appropriate acetophenones (2.72 mmol) in ethanol (30 mL) is stirred at room temperature for 3–4 h. The formed precipitate is collected by filtration, washed with ethanol and crystallized from ethanol.

#### 6-[2-(1-Phenylethylidene)hydrazino]-1-propylpyrimidine-2,4(1*H*,3*H*)-dione (**7a**)

Yield: 83%; m.p. = 207–208 °C; IR (KBr) ν_max_ (cm^−1^): 3154 (NH), 3052 (CH arom.), 2963, 2870 (CH aliph.), 1715, 1691 (C=O), 1602 (C=N), 1499 (C=C); ^1^H-NMR (DMSO-*d*_6_): 11.06 (s, 1H, NH), 8.96 (s, 1H, NH), 7.90–7.82 (dd, 2H, *J* = 9.2 Hz, H_arom_), 7.44–7.43 (m, 3H, arom.), 5.39 (s, 1H, CH-5), 3.92–3.90 (t, 2H, CH_2_), 2.37 (s, 3H, α-CH_3_), 1.68–1.63 (m, 2H, CH_2_), 0.93–0.87 (t, 3H, CH_3_); ^13^C-NMR (DMSO-*d*_6_): δ = 166.5, 162.5, 161.1, 151.2, 137.6, 129.6, 128.5, 126.5, 78.8, 43.3, 20.2, 14.3, 11.3 ppm; MS: *m*/*z* (%) = M^+^, 286 (44), 271 (44), 257 (21), 167 (36), 159 (64), 158 (64), 144 (45), 131 (40), 124 (20), 120 (100), 118 (48), 104 (72), 103 (38), 96 (21), 78 (30), 77 (96); Anal. calcd. for C_15_H_18_N_4_O_2_ (286.32): C, 62.92; H, 6.34; N, 19.57. Found: C, 63.14; H, 6.39; N, 19.71.

#### 6-[2-{1-[4-(Dimethylamino)phenyl]ethylidene}hydrazino]-1-propylpyrimidine-2,4 (1*H*,3*H*)-dione (**7b**)

Yield: 79%; m.p. = 258–260 °C; IR (KBr) ν_max_ (cm^−1^): 3188 (NH), 3069 (CH arom.), 2961 (CH aliph.), 1704 (C=O), 1593 (C=N), 1504 (C=C), 858 (*p*-substituted phenyl); ^1^H-NMR (DMSO-*d*_6_): 11.11 (s, 1H, NH), 9.13 (s, 1H, NH), 8.29–8.26 (d, 2H, *J *= 8.8 Hz, H_arom_), 8.16–8.13 (d, 1H, *J* = 8.8 Hz, H_arom_), 8.10–8.08 (d, 1H, *J* = 8.8 Hz, H_arom_), 5.48 (s, 1H, CH-5), 4.02–3.94 (t, 2H, CH_2_), 3.29 (s, 6H, 2CH_3_), 2.42 (s, 3H, α-CH_3_), 1.68–1.64 (m, 2H, CH_2_), 0.94–0.89 (t, 3H, CH_3_); ^13^C-NMR (DMSO-*d*_6_): δ = 166.5, 162.5, 159.7, 151.1, 150.9, 144.0, 127.3, 123.6, 79.8, 43.4, 42.4, 20.2, 14.2, 11.3 ppm; MS: *m*/*z* (%) = M^+^, 329 (2), 302 (100), 289 (8), 274 (21), 203 (34), 149 (21), 124 (12), 117 (55), 96 (21); Anal. calcd. for C_17_H_23_N_5_O_2_ (329.39): C, 61.99; H, 7.04; N, 21.26. Found: C, 62.12; H, 7.18; N, 21.49.

#### 5-[(Dimethylamino)methylene]-1-propylpyrimidine-2,4,6(1*H*,3*H*,5*H*)-trione 6-{[1-phenylethylidene]hydrazone} (**8**)

##### Method A

A solution of 6-[2-(1-phenylethylidene)hydrazino]-1-propylpyrimidine-2,4(1*H*,3*H*)-dione (**7a**) (0.7 mmol) in dimethylformamide-dimethylacetal (4 mL) is heated under reflux for 1 h. The reaction mixture is evaporated under reduced pressure. The residue is treated with ethanol (10 mL); the formed precipitate is filtered, washed with ethanol, and crystallized from DMF/ethanol (1:3) to afford compound **8**.

##### Method B

A solution of 6-[2-(1-phenylethylidene)hydrazino]-1-propylpyrimidine-2,4(1*H*,3*H*)-dione (**7a**) (0.7 mmol) in dimethylformamide-dimethylacetal (1.5 mL) and DMF (1.5 mL) is heated under reflux for 15 min. The reaction mixture is evaporated under reduced pressure. The residue is treated with ethanol (10 mL); the formed precipitate is filtered, washed with ethanol, and crystallized from DMF/ethanol (1:3) to afford compound **8**.

Yield: method A 90%, method B 94%; m.p. = 220–221 °C; IR (KBr) ν_max_ (cm^−1^): 3136 (NH), 3030 (CH arom.), 2952, 2866 (CH aliph.), 1690, 1656 (C=O), 1562 (C=N), 1512 (C=C); ^1^H-NMR (DMSO-*d*6): 10.13 (s, 1H, NH), 8.08 (s, 1H, CH), 7.73–7.71 (d, 2H, *J *= 7.6 Hz, H_arom_), 7.45–7.36 (m, 3H, arom.), 3.97–3.94 (t, 2H, CH_2_), 3.19 (s, 3H, N-CH_3_), 3.05 (s, 3H, N-CH_3_), 2.39 (s, 3H, CH_3_), 1.69–1.64 (m, 2H, CH_2_), 0.92–0.88 (t, 3H, CH_3_); ^13^C-NMR (DMSO-*d*_6_): δ = 163.8, 159.3, 155.0, 153.6, 151.0, 138.5, 128.6, 128.3, 125.4, 83.0, 46.2, 42.9, 42.7, 20.5, 14.0, 11.3 ppm; MS: *m*/*z* (%) = M^+^, 341 (100), 325 (35), 297 (63), 296 (49), 160 (37), 123 (30), 103 (42), 91 (24), 77 (42), 42 (24); Anal. calcd. for C_18_H_23_N_5_O_2_ (341.40): C, 63.32; H, 6.79; N, 20.51. Found: C, 62.95; H, 7.34; N, 20.39.

#### 5-Methyl-2-(1-phenylvinyl)-7-propyl-2*H*-pyrazolo[3,4-d]pyrimidine-4,6(5*H*,7*H*)-dione (**9**)

##### Method A

A solution of 6-[2-(1-phenylethylidene) hydrazino]-1-propyl-pyrimidine-2,4(1*H*,3*H*)-dione (**7a**) (1.05 mmol) in dimethylformamide-dimethyl acetal (1.5 mL) and DMF (1.5 mL) is heated under reflux for 1 h. The reaction mixture is evaporated under reduced pressure. The residue is treated with ethanol (10 mL), the formed precipitate is filtered, washed with ethanol, and crystallized from DMF/ethanol (1:3) to afford compound **9**.

##### Method B

A mixture of 6-[2-(1-phenylethylidene)hydrazino]-1-propyl- pyrimidine-2,4(1*H*,3*H*)-dione (**7a**) (1.05 mmol) and dimethylformamide-dimethylacetal (3 mL) is heated under reflux for 12 h. The reaction mixture is evaporated under reduced pressure. The residue is treated with ethanol (10 mL), the formed precipitate is filtered, washed with ethanol, and crystallized from DMF/ethanol (1:3) to afford compound **9**.

##### Method C

A mixture of 5-[(dimethylamino)methylene]-1-propylpyrimidine-2,4,6(1*H*,3*H*,5*H*)-trione 6-{[1-phenylethylidene]hydrazone} (**8**) (1.17 mmol) and DMF-DMA (3 mL) is heated under reflux for 1 h. The reaction mixture is evaporated under reduced pressure. The residue is treated with ethanol (15 mL), the formed precipitate is filtered, washed with ethanol, and crystallized from DMF/ethanol (1:3) to afford compound **9**.

Yield: method A 82%, method B 74%, method C 92%; m.p. = 148–149 °C; IR (KBr) ν_max_ (cm^−1^): 3095 (CH arom.) 2950, 2875 (CH aliph.), 1760, 1701 (C=O), 1591 (C=N), 1546 (C=C); ^1^H-NMR (DMSO-*d*6): 8.60 (s, 1H, CH-3), 7.44–7.36 (m, 5H, arom.), 5.71–5.61 (dd, 2H, =CH_2_), 3.87–3.83 (t, 2H, CH_2_), 3.23 (s, 3H, CH_3_), 1.69–1.64 (m, 2H, CH_2_), 0.86–0.83 (t, 3H, CH_3_); ^13^C-NMR (DMSO-*d*6): δ = 157.8, 151.0, 150.3, 144.6, 134.2, 131.8, 129.5, 128.5, 127.3, 109.0, 101.7, 44.7, 27.6, 20.2, 11.0 ppm; MS: *m*/*z* (%) = M^+^, 310 (100), 268 (75), 267 (54), 224 (65), 122 (64), 103 (67),77 (34); Anal. calcd. for C_17_H_18_N_4_O_2_ (310.35): C, 65.79; H, 5.85; N, 18.05. Found: C, 66.01; H, 5.89; N, 18.24.

#### 2,5-Dimethyl-7-propyl-2*H*-pyrazolo[3,4-*d*]pyrimidine-4,6(5*H*,7*H*)-dione (**11**)

A solution of 6-hydrazinyl-1-propyluracil (**4**) (1.63 mmol) in dimethylformamide-dimethylacetal (1.5 mL) and DMF (1.5 mL) is heated under reflux for 1 h. The reaction mixture is evaporated under reduced pressure. The residue is treated with ethanol (10 mL), the formed precipitate is filtered, washed with ethanol, and crystallized from DMF/ethanol (1:3) to afford compound **11**.

Yield: 88%; m.p. = 167–169 °C; IR (KBr) ν_max_ (cm^−1^): 3094 (CH arom.), 2951, 2877 (CH aliph.), 1751, 1698 (C=O), 1586 (C=N), 1544 (C=C); ^1^H-NMR (DMSO-*d*_6_): 8.41 (s, 1H, CH-3), 3.88 (s, 1H, N(2)-CH_3_), 3.87–3.83 (t, 2H, CH_2_), 3.19 (s, 3H, N(5)-CH_3_), 1.70–1.65 (m, 2H, CH_2_), 0.90–0.86 (t, 3H, CH_3_); ^13^C-NMR (DMSO-*d*_6_): δ = 157.9, 151.0, 149.8, 131.8, 100.1, 44.7, 40.4, 27.5, 20.3, 11.0 ppm; MS: *m*/*z* (%) = M^+^, 222 (30), 180 (52), 135 (100), 123 (28), 42 (15); Anal. calcd. for C_10_H_14_N_4_O_2_ (222.24): C, 54.04; H, 6.35; N, 25.21. Found: C, 54.13; H, 6.43; N, 25.45.

## Biological investigation

### Evaluation of the antitumor activity

#### Mammalian cell lines

The cell line that used in this study was human lung carcinoma cell line (A549 cells) is obtained from tissue culture Unit, VACSERA, Cairo, Egypt.

The mammalian cells are propagated in Dulbecco’s modified Eagle’s [41] medium (DMEM) or RPMI-1640 depending on the type of cell line supplemented with 10% heat-inactivated fetal bovine serum, 1% l-glutamine, HEPES buffer and 50 µg/mL gentamycin. All cells are maintained at 37 °C in a humidified atmosphere with 5% CO_2_ and are subcultured two times a week along experimentation.

### i-Antitumor activity evaluation using viability assay

Antitumor activity assay is carried out according to the method described literature [42]. All the experiments concerning the cytotoxicity evaluation are performed and analyzed by tissue culture unit at the regional center for mycology and biotechnology RCMB, Al-Azhar University, Cairo, Egypt.

#### Procedure

The A549 tumor cells are seeded in 96-well plate in 100 µL of growth medium at a cell concentration of 1 × 10^4^ cells/well. After 24 h of seeding, the monolayers are then washed with sterile phosphate buffered saline (0.01 M pH 7.2) and simultaneously the cells are treated with 100 µL from different dilutions of the test sample in fresh maintenance medium and incubated at 37 °C. Different two-fold dilutions of the tested compound (started from 500 to 0.25 µM) are added to confluent cell monolayers dispensed into 96-well, flat-bottomed microtiter plates (Falcon, NJ, USA) using a multichannel pipette. The microtiter plates are incubated at 37 °C in a humidified incubator with 5% CO_2_ for a period of 72 h. Untreated cells are served as controls. Three independent experiments are performed each containing six replicates for each concentration of the tested samples. The cytotoxic effects of the tested compounds are then measured using crystal violet staining viability assay. Briefly, after 72 h of treatment, the medium is removed, 100 μL of 0.5% of crystal violet in 50% methanol is added to each well and incubated for 20 min at room temperature and subsequently excess dye is washed out gently by distilled water. The plate is allowed to dry then the viable crystal violet-stained cells are lysed using 33% glacial acetic acid solution. Absorbance at 570 nm is then measured in each well using microplate reader (Sunrise, TECAN, Inc, USA). Toxoflavin and 5-fluorouracil are used as positive control. The absorbance is proportional to the number of surviving cells in the culture plate.

## Conclusions

A series of newly synthesized compounds of substituted benzaldehyde-pyrimidin-4-yl)hydrazones (**5a**–**f**), pyrimido[5,4-*e*][1,2,4]triazines **6a**–**e**, arylethylidenehydrazinylpyrimidines **7a**,**b** and pyrazolopyrimidines **9**,**11** are prepared via a simple method starting from the substrate 6-hydrazinyl-1-propyluracil (**4**). The synthesized compounds exhibited good cytotoxic activity against human lung carcinoma (A549) cell line and the highest effect is measured for compound **6b** with IC_50_ value 3.6 μM, followed by compounds **9**, **5a**, **8**, **5e**, **6e**, **5b**, **5f**, **7a**, **5c**, **6c**, **7b**, **6a**, **11**, **5d** and **6d** with IC_50_ values of 26.3, 26.8, 28.4, 49.3, 53.8, 54.7, 60.2, 60.5, 74.3, 81.5, 104.6, 107.1, 123, 238.7, and 379.4 μM, compared with reference drug 5-fluorouracil (10.5 μM).
